# Maternal high fat diet induces circadian clock-independent endocrine alterations impacting the metabolism of the offspring

**DOI:** 10.1016/j.isci.2024.110343

**Published:** 2024-06-21

**Authors:** Lu Ding, Benjamin D. Weger, Jieying Liu, Liyuan Zhou, Yenkai Lim, Dongmei Wang, Ziyan Xie, Jing Liu, Jing Ren, Jia Zheng, Qian Zhang, Miao Yu, Meltem Weger, Mark Morrison, Xinhua Xiao, Frédéric Gachon

**Affiliations:** 1 Key Laboratory of Endocrinology of National Health Commission, Diabetes Research Center of Chinese Academy of Medical Sciences, Department of Endocrinology, Peking Union Medical College Hospital, Peking Union Medical College, Chinese Academy of Medical Sciences, Beijing 100730, China; 2 Institute for Molecular Bioscience, The University of Queensland, St. Lucia, QLD 4072, Australia; 3 Department of Medical Research Center, Peking Union Medical College Hospital, Chinese Academy of Medical Sciences & Peking Union Medical College, Beijing 100730, China; 4 Department of Endocrinology, Beijing Chaoyang Hospital, Capital Medical University, Beijing 100022, China; 5 Frazer Institute, Faculty of Medicine, The University of Queensland, Woolloongabba, QLD 4102, Australia; 6 Department of Endocrinology, Peking University First Hospital, Beijing 100034, China; 7 Australian Infectious Diseases Research Centre, St. Lucia, QLD 4072, Australia

**Keywords:** neuroscience, behavioral neuroscience, molecular neuroscience, omics, transcriptomics

## Abstract

Maternal obesity has long-term effects on offspring metabolic health. Among the potential mechanisms, prior research has indicated potential disruptions in circadian rhythms and gut microbiota in the offspring. To challenge this hypothesis, we implemented a maternal high fat diet regimen before and during pregnancy, followed by a standard diet after birth. Our findings confirm that maternal obesity impacts offspring birth weight and glucose and lipid metabolisms. However, we found minimal impact on circadian rhythms and microbiota that are predominantly driven by the feeding/fasting cycle. Notably, maternal obesity altered rhythmic liver gene expression, affecting mitochondrial function and inflammatory response without disrupting the hepatic circadian clock. These changes could be explained by a masculinization of liver gene expression similar to the changes observed in polycystic ovarian syndrome. Intriguingly, such alterations seem to provide the first-generation offspring with a degree of protection against obesity when exposed to a high fat diet.

## Highlights

• Maternal obesity has little impact on the rhythmic physiology of the offspring
• The impact on the microbiome composition appears mainly driven by feeding
• Liver inflammation and mitochondrial activity are impacted by maternal obesity
• This likely results from the masculinization of the offspring similar to PCOS models

## Graphical abstract

**Figure:**
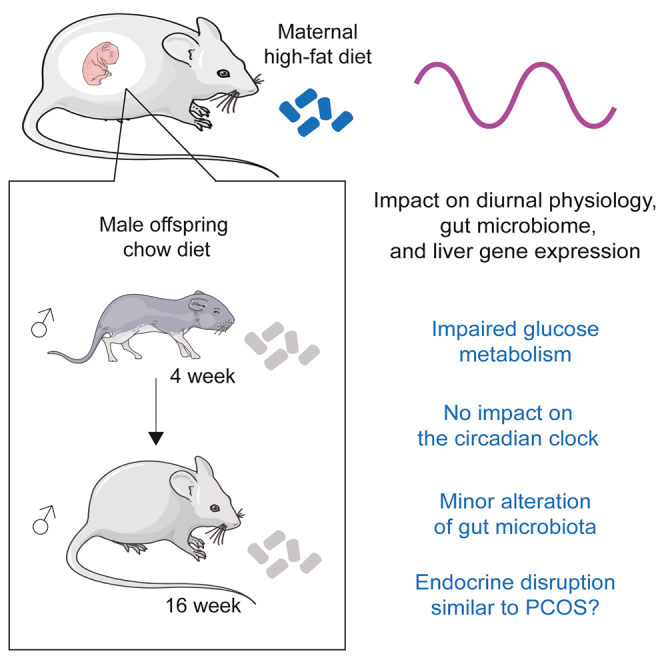

## Teaser

Circadian rhythms; Behavioral neuroscience; Molecular neuroscience; Omics; Transcriptomics; Endocrinology.

## Introduction

The global obesity epidemic has resulted in a rise in metabolic disorders among women of reproductive age. Currently, about 30–50% of women of reproductive age falling into the overweight or obese range.^1^^,^^2^ This rise has also profound implications for offspring metabolism spanning from the fetal stage to adulthood.^3^^,^^4^^,^^5^ Both epidemiological and animal studies have shown that maternal high fat diet (HFD) and obesity increase the offspring’s risk of developing diabetes, obesity, and cardiometabolic diseases.^6^^,^^7^^,^^8^^,^^9^ This phenomenon of metabolic imprinting has given rise to the concept of Developmental Origins of Health and Disease (DOHaD).^10^^,^^11^

To uncover the mechanisms behind this transgenerational effect, recent hypotheses propose an intergenerational alteration of the interplay between gut microbiota and the circadian clock. The circadian clock is an endogenous oscillator that orchestrates most aspects of mammalian physiology and behavior in the anticipation of environmental day-night changes. This includes the sleep/wake cycle, feeding/fasting cycles, and rhythmic hormone secretion. Organized in a hierarchical manner, the central circadian clock localized in the suprachiasmatic nuclei (SCN) of the hypothalamus synchronizes peripheral clocks present in virtually every cell of the organism.^12^^,^^13^ While the central clock is synchronized by light through direct connections between the retina and the SCN, peripheral clocks can be also synchronized by feeding and other systemic cues.^14^ The importance of the circadian clock for physiology and metabolism is underscored by its disruption in scenarios such as shift work, that lead to metabolic diseases.^15^^,^^16^ Conversely, HFD and obesity have been observed to disrupt the circadian clock.^17^^,^^18^ Accordingly, a few studies have reported a transgenerational impact of maternal HFD and obesity on the circadian rhythm of the offspring in rodents and primates.^19^^,^^20^^,^^21^^,^^22^

The feeding/fasting cycle not only synchronizes peripheral clocks but also leads to changes in daily fluctuations in the composition of the gut microbiota, further influencing host physiology.^23^^,^^24^ Given that obesity is known to impact the gut microbiome and is associated with dysbiosis,^25^ alterations in gut microbiota composition are suggested to mediate the metabolic consequences of maternal HFD.^26^^,^^27^^,^^28^ A disrupted rhythmic gut microbiome could also potentially impact the diurnal physiology of the offspring. For these reasons, we explored whether the metabolic effects of maternal HFD on offspring could be attributed to disturbances in rhythmic gut microbiota and liver physiology. While the liver circadian clock and feeding rhythms appear not impacted by maternal HFD, we observed alterations in the rhythmic expression of genes involved in pathways related to mitochondrial activity or ribosome biogenesis. Further analysis revealed an increased expression of genes involved in pathways related to inflammation, potentially associated with perturbations in endocrine regulations. These findings could help to elucidate the transgenerational effects of maternal HFD on offspring physiology.

## Results

### Maternal high fat diet induces glucose and cholesterol metabolism alterations in the offspring

**Figure 1:**
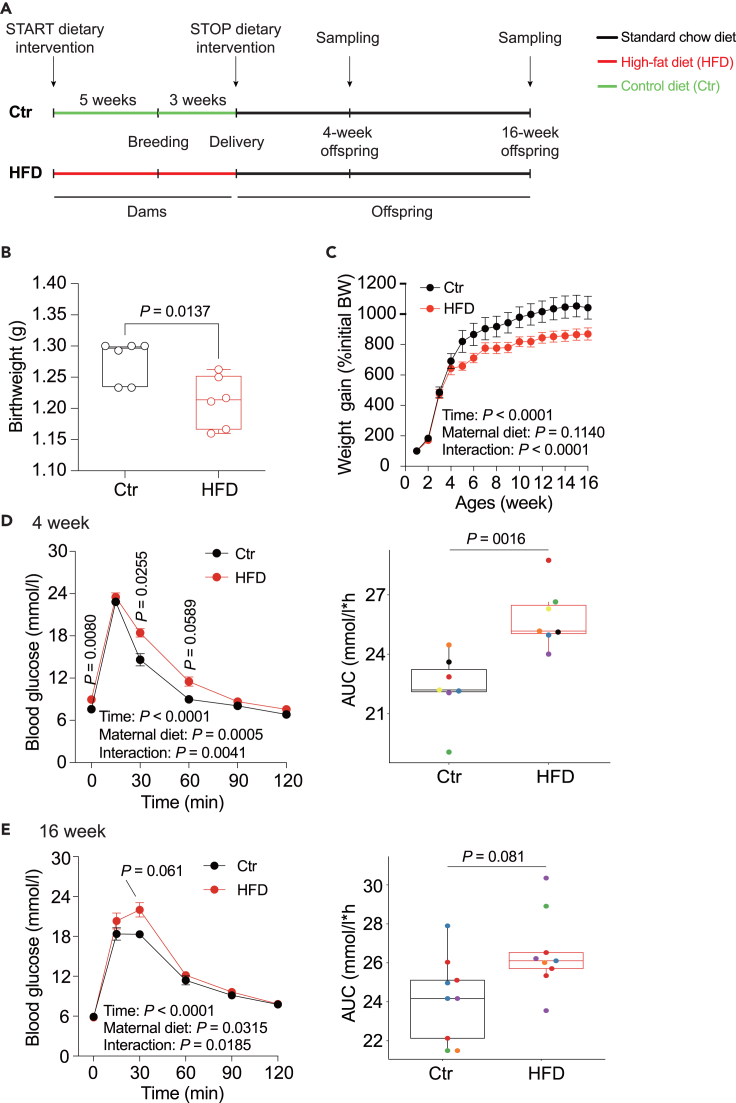
Maternal HFD impacts glucose and cholesterol metabolism in offspring (A) Experimental setup assessing the effects of maternal HFD before and throughout pregnancy on metabolic and circadian changes in offspring. (B) Offspring birthweight. (C) Percentage change in offspring body weight throughout the study. (D and E) Glucose tolerance test (left) and the corresponding area under the curve (right, each color represents different litter) for 4-week-old offspring (D) and 16-week-old offspring (E). N = 5–6 mice (from different litters) per experimental group. All boxplots are Tukey boxplots and data is assessed with a Student’s t-test; line chart data are presented as mean ± S.E.M. and are analyzed via a repeated measure two-way ANOVA or mixed linear model followed by Šídák post hoc tests. The details of the statistical analysis results are available in Table S6. Ctr: maternal control diet (black); HFD: maternal high-fat diet (red).

To determine the impact of maternal HFD on offspring metabolism, we established an intergenerational mouse model by feeding female mice a control (Ctr) diet or HFD starting 5 weeks before mating and throughout pregnancy. The induced metabolic phenotype caused by this treatment on the dams was described elsewhere.^29^ All dams and litters were transferred to a normal chow diet after delivery. To avoid nutritional bias between litters, the litter sizes were homogenized to 6 pups for each dam within the first 3 post-natal days. Male offspring were weaned at postnatal day 21 and fed a standard chow diet until 16 weeks of age (Figure 1A). The results showed that maternal HFD results in reduced body weight of the offspring at birth (Figure 1B). This phenotype is commonly seen in studies using similar mouse models but also exhibits high experimental variability.^8^^,^^30^ While body weight gain was not statistically different between animals from Ctr or HFD fed mothers, there was a significant interaction between time and maternal diet and animals from HFD mothers were lighter throughout the experiment (Figure 1C). While neither liver weight of the offspring at 4 and 16 weeks (Figure S1A) nor liver triglyceride concentration were statistically different, we observed an increase in liver cholesterol concentration in 16-week-old offspring (Figures S1B and S1C). We also did not detect significant alterations in serum total cholesterol (TC), triglycerides (TG), free fatty acids (FFAs), high-density lipoprotein cholesterol (HDL-C), low-density lipoprotein cholesterol (LDL-C), alanine transaminase (ALT), and aspartate aminotransferase (AST) in the offspring, but observed a significant increase in glucose levels at 4 weeks, suggesting impaired glucose tolerance (Figures S2).

Contrary to previous studies suggesting that glucose tolerance is often unaltered when a Ctr diet is provided after birth,^31^^,^^32^^,^^33^ our data revealed a slightly delayed and impaired glucose tolerance in the offspring. This is evidenced by elevated blood glucose levels 30 min post glucose load and a larger AUC at 4 weeks and 16 weeks compared to the Ctr group (Figure 1D and 1E). These findings are similar to studies that maintained HFD during lactation.^34^^,^^35^ However, there were no significant changes in insulin tolerance (Figures S1D and S1E). Taken together, the data suggest that prenatal exposure to maternal HFD, followed by a control chow diet postnatally, slightly impairs glucose and cholesterol metabolism, but overall physiology appears largely unaffected, aligning with prior findings.^31^^,^^32^^,^^33^

### Diurnal rhythms in food intake and energy expenditure are subtly altered by maternal high fat diet

**Figure 2:**
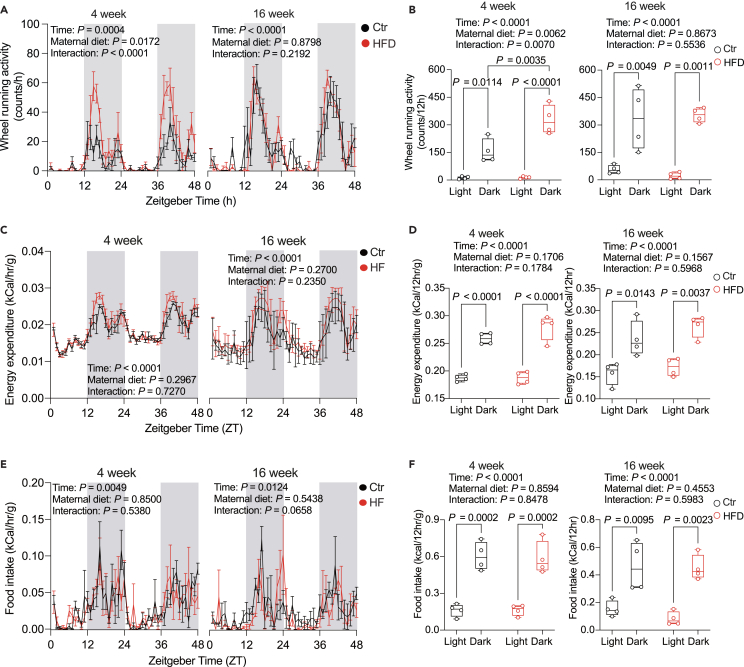
Maternal HFD marginally affects diurnal food consumption and energy expenditure (A, C, and E) Diurnal rhythms of running wheel activity (A), energy expenditure (C), and food intake (E) in 4-week-old (left) and 16-week-old offspring (right). (B, D and F) Daily alterations in running wheel activity (B), energy expenditure (D), and food intake (F) in 4-week-old (left) and 16-week-old offspring (right). Zeitgeber time (ZT) indicates light entrainment periods (ZT0-12: lights on; ZT12-24: lights off). *N* = 4 mice (from different litters) per group. All boxplots are Tukey boxplots and line chart data are presented as mean ± S.E.M. Data are analyzed via a repeated measure two-way ANOVA followed by Šídák post hoc tests. The details of the results of the statistical analyses are available in Table S6. Ctr: maternal control diet (black); HFD: maternal high-fat diet (red).

Mice fed an HFD exhibit attenuated rhythms of the circadian clock and feeding rhythms.^18^^,^^36^ A previous study linked gestational obesity with altered daily rhythms of activity and food intake in 15 weeks old offspring.^20^ We thus measured the diurnal rhythms of running wheel locomotor activity, energy expenditure, and feeding in the offspring of Ctr and HFD dams at 4 and 16 weeks of age. At 4 weeks, we observed an increase in total locomotor activity and a mild but not significant increase in energy expenditure. However, these differences were not present at 16 weeks (Figure 2A–2D), diverging from a previous report.^20^ In addition, there were no observed differences in feeding rhythms (Figures 2D and 2E), suggesting that metabolic alterations were not associated with changes in diurnal feeding behavior.

### Maternal high fat diet modifies the rhythmic gut microbiome of the offspring

Given that maternal HFD before and during pregnancy leads to changes in the rhythmic cecal microbiome composition of the dams^29^ and microbiome alterations are transmitted to the offspring,^27^^,^^37^ we aimed to investigate potential alterations in the rhythmic gut microbiome of the offspring through the analysis of 16S rRNA gene amplicon profiles. At the global level, α-diversity analysis revealed that the expected altered Simpson index diversity observed in dams under HFD was still present in 4-weeks old offspring, albeit less pronounced. However, this difference disappears in 16-weeks old offspring, suggesting that the Ctr diet after birth normalized the altered diversity caused by maternal HFD (Figure S3A). Accordingly, while the Shannon diversity index was also different in dams, this difference disappears in the offspring (Figure S3A). Overall, the 16S rRNA analyses revealed that microbiota communities were dominated by *Firmicutes* in all mice and time points (Figure S3B and Table S1) and that the difference in gut microbiota abundance observed in HFD fed dams is normalized under Ctr diet after birth.

**Figure 3:**
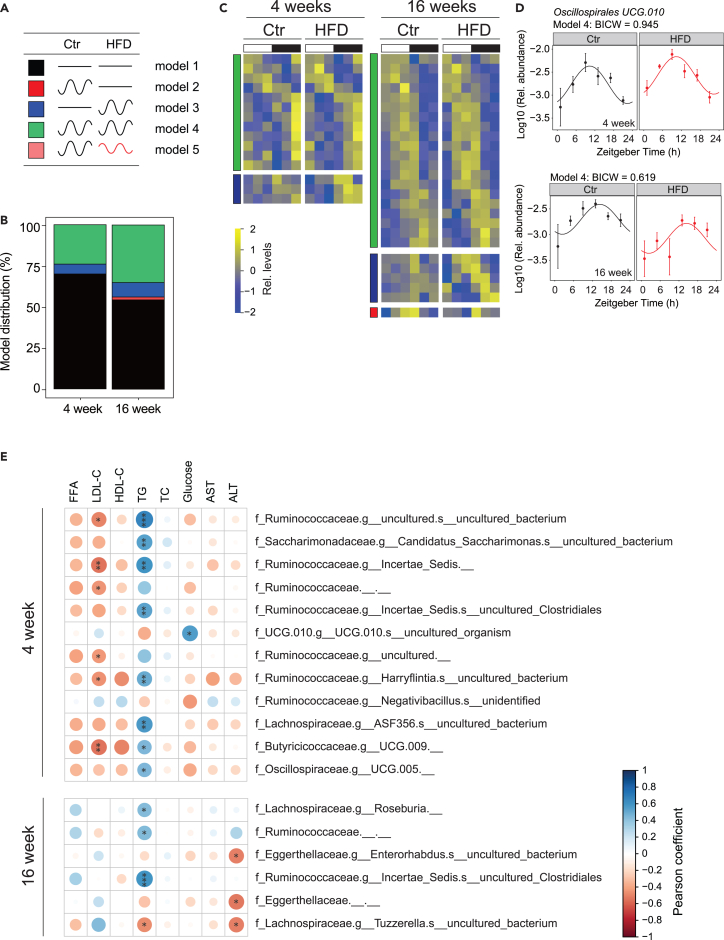
Maternal exposure to HFD alters gut microbiome composition in offspring (A) Model selection for rhythmic ASVs in male offspring from maternal HFD and maternal Ctr groups: black line, non-rhythmic ASVs; black sinus wave: rhythmic ASVs; red sinus wave, rhythmic ASVs with different phase and/or amplitude. (B) Model distribution percentage of ASVs across models 1–5, with different colors indicating the respective model as illustrated in (A). (C) Heatmap showing rhythmic ASVs in 4-week-old (left) and 16-week-old offspring (right). Standardized relative ASV abundance is indicated in blue (low) and yellow (high). The white and black bars denote light conditions. Different color indicates the corresponding model as shown in (A). (D) Exemple of rhythmic ASV in 4-week-old (top) and 16-week-old offspring (bottom). Each dot represents the mean ASV abundance for each zeitgeber time (ZT) with the line illustrating the cosinor regression fit. The ZT defines the timing of entrainment by light (ZT0: lights on; ZT12: lights off). (E) Correlation plots based on Pearson coefficient between serum metabolic profiles and ASV abundance. Only correlations with a Pearson coefficient that had an associated Benjamini-Hochberg adjusted *p*-value of less than 0.05 (determined through Fisher’s Z transform) were deemed statistically significant. The details of the results of the statistical analyses are available in Table S6. Colors represent positive (blue) and negative (red) correlation. Size of the circles indicates the corresponding *p*-value. *N* = 4 mice (from different litters) per group. Ctr: maternal control diet (black); HFD: maternal high-fat diet (red).

We employed *dryR*^38^ to analyze the rhythmic amplicon sequence variants (ASV) identified in the 16S profiles that were among the ones present in at least 50% of samples in Ctr and HFD offspring. This resulted in the characterization of five models defined by the differential rhythmicity between the groups (Figure 3A and Table S1). At both 4 and 16 weeks, most ASVs were found in model 1 (not rhythmic) and model 4 (similar rhythmicity in Ctr and HFD offspring) (Figures 3B and 3C). This observation aligns with previous studies indicating that feeding rhythm, which shows no difference between Ctr and HFD offspring (Figures 2E and 2F), is the primary driver of microbiome rhythmicity, with only a limited influence from other factors.^39^^,^^40^^,^^41^ Among the rhythmic microbiome, we found that *Oscillospirales* exhibited robust rhythmicity in 4- and 16-week offspring across all feeding conditions (Figure 3D). We identified only a few bacterial groups with different rhythmicity between Ctr and HFD offsprings that globally showed a gain in rhythmicity in the HFD offspring (Figure 3B, Table S1). To determine the association between the microbiome composition and metabolism, we performed a correlation analysis between the relative abundance of each bacterial group with the measured metabolic parameters (Figure 3E). Circulating triglyceride levels were the only parameter that showed a broad correlation with ASVs, likely reflecting that feeding rhythm is the main drivers of the microbiome and triglyceride rhythms.^42^

**Figure 4:**
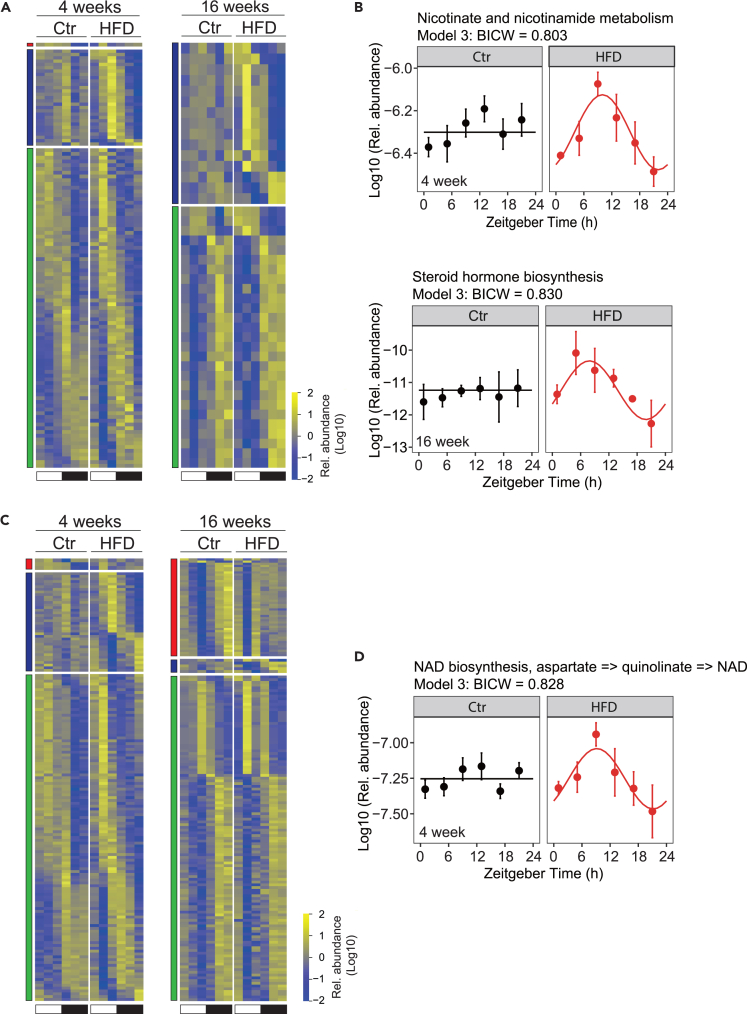
Maternal HFD alters the functional attributes of the offspring’s gut microbiome (A and C) Heatmap for rhythmic KEGG pathway (A) and KEGG module (C) in 4-week-old offspring (left) and 16-week-old offspring (right). Standardized relative pathway/module abundance is indicated in blue (low) and yellow (high). White and black bars indicate light conditions. Different color indicates the corresponding model as shown in Figure 3A. (B) Nicotinate and nicotinamide metabolism pathway in 4-week-old (top) and steroid hormone biosynthesis pathway in 16-week-old offspring (bottom). (D) NAD^+^ biosynthesis module in 4-week-old offspring. The dots mark values of inferred functional activity for each zeitgeber time (ZT) with the line illustrating the cosinor regression fit. The ZT defines the timing of entrainment by light (ZT0: lights on; ZT12: lights off). *N* = 3 mice (from different litters) per group. Ctr: maternal control diet (black); HFD: maternal high-fat diet (red).

Interestingly, a similar rhythmic analysis of the metagenomic data and the predicted metabolic functions of the cecal microbiome showed a slightly different result. While most of the KEGG pathways were still in models 1 and 4, we detected a gain of rhythmicity (model 3) in HFD offspring at both 4 and 16 weeks for some pathways, in agreement with the analysis of 16S rRNA (Figures 4A, S4A, and Table S2). Among the KEGG pathways that showed conserved rhythmicity at both 4 and 16 weeks (model 4) were pathways involved in cell motility or environmental adaptation (Figure S4B), confirming that such general functions are mainly driven by feeding rhythms.^40^ Many of the pathways that gained rhythmicity under a maternal HFD were involved in metabolism, including metabolisms of nicotinamide at 4 weeks and steroid hormones at 16 weeks, potentially influencing in this way the rhythmic host metabolism (Figure 4B). Our observation was different for KEGG modules that showed a gain of rhythmicity at 4 weeks (model 3) but a loss of rhythmicity at 16 weeks (model 2) (Figures 4C, S4C, and Table S2). Among those modules with conserved rhythmicity at 4 and 16 weeks were those involved with hydrogen sulfide (H_2_S) and methane metabolism (Figure S4D). Both these modules can support important hydrogen “sinks” during fermentation: sulfide in particular can be produced and released by some gut bacteria during their metabolism of sulfur-containing dietary proteins and/or other endogenous sulfated compounds (e.g., sulfomucins and/or some secondary bile acids) and has a broad physiological role, including influencing local microbiome.^43^^,^^44^ Interestingly, we also noticed in the HFD offspring at 4 weeks a gain of rhythmicity for nicotinamide adenine dinucleotide (NAD^+^) synthesis which can play a central role in hydrogen (proton) transactions (Figure 4D).

### Impact of maternal high fat diet on liver gene expression

**Figure 5:**
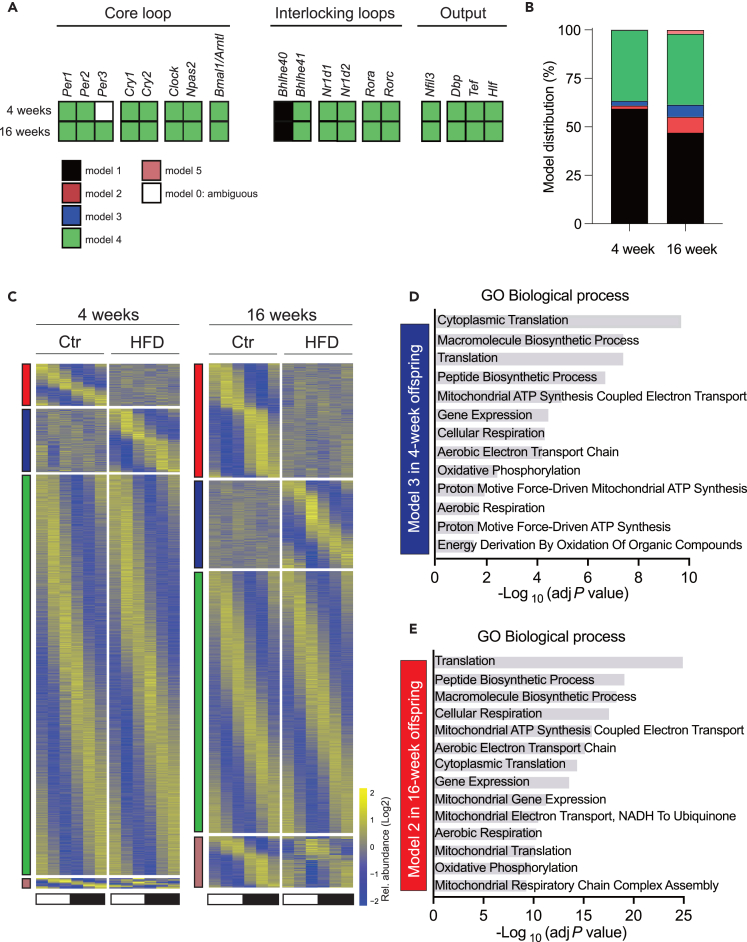
Impact of maternal HFD on rhythmic liver gene expression (A) Hepatic circadian clock genes show an unaltered temporal expression profile under maternal HFD, with most genes assigned to model 4. (B) Model distribution percentage of genes in model 1–5. Different color indicates the corresponding model as shown in (A). (C) Heatmap for rhythmic genes in 4-week-old offspring (left) and 16-week-old offspring (right). Standardized relative gene expression is indicated in blue (low) and yellow (high). White and black bars indicate light conditions. Different color indicates the corresponding model as shown in (A). (D and E) Enrichment of GO biological process for genes in model 3 in 4-week-old offspring (D) and in model 2 in 16-week-old offspring (E). N = 2–3 mice (from different litters) per group. Ctr: maternal control diet (black); HFD: maternal high-fat diet (red).

To gain more insight into the impact of maternal HFD on rhythmic liver gene expression, we quantified mRNA by RNA sequencing and analyzed differential rhythmic gene expression with *dryR*. A focused analysis of the rhythmic expression of the core circadian clock genes shows that the temporal expression profiles of liver circadian clock genes are globally unaffected by maternal obesity (Figures 5A, S5A, and Table S3), in contrast to previous reports.^19^^,^^22^ A more global analysis of rhythmicity shows that most of the rhythmic genes exhibited a similar rhythmicity in Ctr and HFD mice at both 4 and 16 weeks (Figures 5B and 5C). Nevertheless, more differentially expressed genes were found in 16-week-old mice compared to 4-week-old. Among all the genes that showed rhythmicity, 15.6 and 11.7% of genes showed a loss (model 2) or gain (model 3) of rhythmicity, respectively, at 16 weeks compared to 4.1 and 6.0% at 4 weeks. 0.2 and 3.7% of genes also showed differential rhythmicity (model 5, altered acrophase and/or amplitude) at 4 and 16 weeks, respectively. Interestingly, at 4 weeks, we found enrichment for biological processes only among the genes that gain rhythmicity (Figure 5D, Table S4). Most of these enriched genes are involved in ribosome biogenesis and mitochondrial function (Figure S5B), processes regulated by the circadian clock and feeding rhythms.^45^^,^^46^^,^^47^ Surprisingly, the exact same pathways were enriched among the genes that lost rhythmicity at 16 weeks (Figures 5E, S5C, and Table S4). Interestingly, both ribosome biogenesis^48^^,^^49^ and mitochondrial activity^50^^,^^51^^,^^52^ are regulated by NAD^+^-dependent SIRT7 and SIRT3. This observation may align with the rhythmic NAD^+^ metabolism observed through metagenomics at 4 weeks, highlighting the significant role of the microbiome in regulating host NAD^+^ levels.^53^^,^^54^

**Figure 6:**
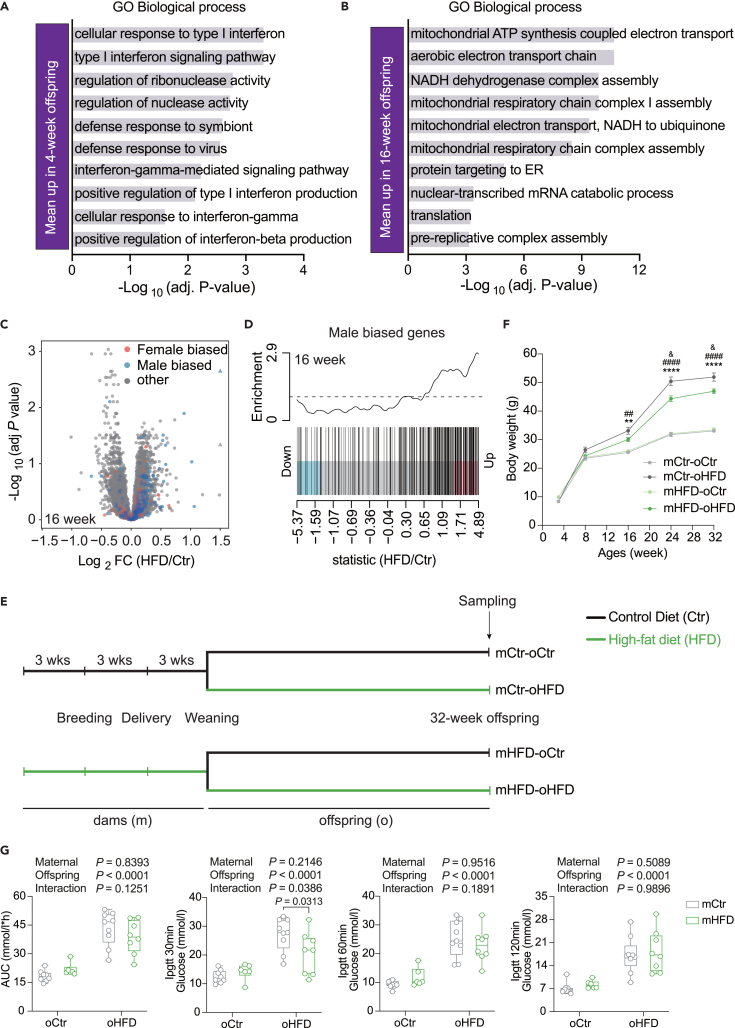
Differential gene expression in offspring exposed to maternal HFD (A and B) GO enrichment analysis of hepatic genes showing a mean increase in expression in 4-week-old (A) and 16-week-old offspring upon maternal HFD (B). (C) Volcano plots illustrating sex-biased differentially expressed genes in the livers of 16-week-old offspring upon maternal HFD. (D) Barcode plots for male-biased genes in the liver of 16-week-old offspring with genes ordered from most down to most upregulated. (E) Experimental design of Zheng et al.^33^ (F) Changes in offspring bodyweight from 4 to 32 weeks of age. (G) Area under the curve for the glucose tolerance test and blood glucose levels at 30-, 60-, and 120-min post-glucose load (2 g/kg) in 32-week-old offspring. N = 2–3 mice (from different litters) per group. All boxplots are Tukey boxplots and line chart data are presented as mean ± S.E.M. Data are analyzed via two-way ANOVA followed by Holm-Šídák post hoc tests. The details of the results of the statistical analyses are available in Table S6. ∗∗*p* < 0.05 and ∗∗∗∗*p* < 0.0001, mHF-oHF vs. mHF-oCtr; ^##^*p* < 0.01 and ^####^*p* < 0.0001, mCtr-oHF vs. mCtr-oCtr; & *p* < 0.05, mCtr-oHF vs. mHF-oHF. mCtr, maternal control diet; mHFD, maternal high-fat diet; oCtr, offspring control diet; oHFD, offspring high-fat diet.

Focusing on differential gene expression at the mean level, upregulated genes at 4 weeks showed a strong enrichment for the type I interferon (IFN) pathway and genes upregulated at 16 weeks exhibited a strong enrichment in mitochondria-associated processes (Figures 6A, 6B, S6A–S6C, Tables S3, and S4). This correlates with a transcriptional signature associated with the IFN-activated IRF9 and STAT1/2 transcription factors at 4 weeks (Figure S6D, Table S5). Previous studies reported that the activation of IFN signaling decreases mitochondrial activity and gene expression and induces mitochondrial stress,^55^^,^^56^ a process that protects against HFD induced obesity^57^^,^^58^^,^^59^ and alters mitochondrial gene expression.^60^^,^^61^ This could therefore constitute an adaptation process to protect against HFD induced obesity.

At 16 weeks, we noticed a transcriptional signature corresponding to the activation of the SUZ12 transcription factor. SUZ12 is one of the three components of the polycomb repressive complex-2 (PRC2)^62^ that plays a role in the liver sex-biased gene expression.^63^ Sex-biased liver gene expression develops after puberty and is regulated by the interaction between growth hormone (GH) and sex hormones.^64^^,^^65^^,^^66^ Therefore, we investigated the sex-biased gene expression and identified a striking increase in male-biased gene at 16 weeks (Figures 6C and 6D). Interestingly, a similar analysis of differential gene expression from a related study using female mice^67^ revealed a similar increase in GH-male induced genes associated with a similar increase of IFN-regulated genes (Figure S6F and Table S5). Therefore, it is plausible that maternal obesity impacts these pathways, influencing in this way the sexual development and fertility of the offspring.^68^^,^^69^ Interestingly, similar sexual development phenotypes are observed in the offspring of female with polycystic ovary syndrome (PCOS),^70^^,^^71^^,^^72^ a condition defined by a combination of androgen excess and ovarian dysfunction from complex etiology.^73^ We therefore analyzed the liver gene expression of a mouse model of PCOS^74^ and found a similar masculinization phenotype of liver gene expression, with an increase of GH male-induced genes and a decrease of GH male repressed genes (Figure S6G). This suggests that these two phenomena, despite having different etiology, may represent the manifestation of a similar physiological condition.

Sex difference influences mitochondrial activity, that is higher in female,^75^^,^^76^ as well as inflammation, with prepubertal male displaying an increase inflammatory response.^77^ It is therefore conceivable that this “masculinization” plays a role in the observed increase in inflammation and disrupted mitochondrial activity, contributing to the observed glucose intolerance. Nevertheless, because these processes have also been shown to protect against HFD-induced obesity,^57^^,^^58^^,^^59^ we hypothesized that it can somehow protect against metabolic syndrome. To test this assumption, we reanalyzed our previously published study with the goal of elucidating the effects of maternal obesity on the metabolism of the offspring^33^ (Figure 6E). While still obese and showing impaired metabolism when exposed to HFD throughout lactation and weaning, male offspring from obese dams unexpectedly exhibited a lower body weight gain (Figures 6F and S6H), improved glucose tolerance (Figure 6G), and insulin sensitivity^33^ compared to animals from Ctr dams. These results suggest that maternal HFD can confer short-term moderate transgenerational protection against metabolic syndrome through endocrine adaptation.

## Discussion

The present study corroborates that maternal HFD before and during pregnancy alters physiology in male offspring, reinforcing the DOHaD concept. This includes a lower body weight at birth, associated with later metabolic defect.^78^ However, while this phenomenon is often observed in mice,^8^^,^^30^ it is less common in humans where maternal obesity is rather associated with increased body weight at birth.^79^ In contrast to previous publications,^19^^,^^20^^,^^21^^,^^22^ maternal obesity was not associated with a significant alteration of the diurnal circadian clock genes expression and behavior. Nevertheless, our findings are consistent with previous reports showing that changes in diurnal behavior and gene expression are absent when offspring from obese rat dams were nursed by non-obese foster dams during lactation and later switched to standard chow diet post-weaning.^19^ Alterations in the expression patterns of circadian clock genes observed in these studies were consistently associated with obesity in the offspring, while a functional circadian clock correlated with normal body weight.^19^^,^^80^ Therefore, the disruption of the circadian clock in these cases is likely a direct consequence of the observed obesity in the offspring rather than a result of maternal obesity. Indeed, obesity induced by an HFD is associated with disruptions of circadian rhythms and circadian clock genes expression.^18^^,^^36^ However, these alterations are absent in mice protected from HFD induced obesity.^17^^,^^81^ This underscores the notion that maternal HFD during lactation and after weaning is an independent risk factor for offspring health outcomes.^82^ Hence, extending maternal dietary intervention to the lactation period likely obscures the effects caused by *in utero* exposure to maternal HFD. In this study, we restricted maternal HFD to the prenatal period to avoid potential confounding effects of lactational exposure, which is generally associated with greater body weight gain in the offspring.^82^ Our findings suggest that while maternal obesity does not directly impact offspring diurnal feeding behavior, it can predispose offspring to alteration induced by HFD through changes in synaptic connections in the hypothalamus.^83^^,^^84^

Analysis of the 24h-rhythmicity of the microbiome confirms the predominant influence of feeding rhythms on microbiota composition and abundance.^39^^,^^40^^,^^41^ Although dysbiosis is associated with obesity, increasing evidence suggests that these changes are primarily driven by caloric load and food composition.^85^^,^^86^ In our study, all animals were fed an identical standard chow diet and exhibited similar feeding rhythms (Figure 2E and 2F). Under these conditions, we observed that the dysbiosis in 4-weeks old offspring from HFD dams gradually resolves, and by 16 weeks, the microbiome composition is similar across all animals. Similarly, another study found little difference in microbiota diversity in adult offspring^87^ and an analysis of various factors influencing microbiota composition highlighted a significant impact of sex which correlates with the obese phenotype observed only in males.^88^^,^^89^ Interestingly, a study using a cross-fostering paradigm demonstrated that the maternal feeding regimen is the main driver of the offspring’s microbiota composition and diversity,^90^ reinforcing the notion that microbiome diversity is largely dictated by feeding. Nevertheless, we found a gain in rhythmicity for a few bacterial groups and associated functions, such as NAD^+^ metabolism, in HFD offspring. This correlates with altered rhythmicity of NAD^+^-regulated ribosome biogenesis and mitochondrial function in the liver of the offspring. Further research will be required to establish a causal relationship between these two phenomena.

One important observation of the present study includes the activation of the type I interferon pathway in 4-week-old HF offspring and mitochondrial pathway activation at 16 weeks of age. The role of the IFN pathway in obesity is complex. While the inactivation of the type I IFN pathway exacerbates metabolic dysfunction associated with obesity,^91^^,^^92^ the activation of the type II IFN pathway protect against HFD induced obesity by altering mitochondrial activity.^93^^,^^94^ Interestingly, recent studies suggest that the simultaneous activation of both IFN pathway and mitochondrial stress can confer protection against HFD-induced obesity.^57^^,^^58^^,^^59^ This might contribute to the partial resistance observed in male offspring from HFD-fed mothers to HFD induced weight gain and glucose intolerance, potentially due to a predictive adaptive response (PAR).^95^ The PAR hypothesis describes a type of developmental plasticity where early life signals shape the development of a phenotype that helps the offspring to adapt better in their future environment. In light of the PAR hypothesis, male offspring in the present study would benefit from maternal HFD due to a match between the *in utero* environment (maternal HFD) and the likely future environment of postnatal HFD exposure. However, published data demonstrate that mitochondrial dysfunction is a risk factor for liver disease and metabolic dysfunction-associated steatohepatitis^96^ and increases the transgenerational susceptibility to metabolic dysfunction-associated steatohepatitis.^97^ It is therefore likely that the short-term benefits of maternal HFD have long-term detrimental effects. In addition, this transgenerational mitochondrial dysfunction might be a contributing factor to the recently described global decrease in basal energy expenditure over the past thirty years, as documented in the study of a human cohort from the United States and Europe.^98^

Another striking observation of the present study is the “masculinization” of liver gene expression of 16 weeks offspring that displays some similarities with a PCOS mouse model. Similar to what is detected in animal models of maternal obesity^8^^,^^30^ or *in utero* exposure to testosterone,^99^^,^^100^ offspring from PCOS women show a lower gestational body weight^101^ that correlates with maternal testosterone levels.^102^ Offspring from PCOS females also show similar metabolic dysfunction^70^^,^^72^ and perturbed sexual development and fertility,^71^^,^^72^ raising the question about what could explain these similarities between maternal obesity and PCOS. Among the different animal models of PCOS,^103^ prenatal exposure to testosterone in primates,^104^ sheep,^105^ mice,^106^ and rats^107^ induces phenotypes similar to PCOS, associated with an increased circulating testosterone level in the offspring. Interestingly, although testosterone levels rise during pregnancy in humans,^108^ this surge in testosterone is more pronounced during the last trimester of pregnancy in obese women^109^ and obese rats.^110^ Therefore, it appears that high testosterone during the prenatal period of the pregnancy of obese female could play a role in the DOHaD associated with maternal obesity during pregnancy. Additionally, testosterone impacts glucose metabolism^107^ and locomotor activity,^111^^,^^112^ which could explain the observed increases in glucose intolerance (Figure 1D), glucose levels (Figure S2A), and locomotor activity at 4 weeks (Figure 2A). In addition, testosterone exposure *in utero* could play a role in the reported alteration of DNA methylation observed in the offspring of obese mothers,^113^ testosterone inducing DNA demethylation after puberty.^114^ Altogether, these results demonstrate similarities between PCOS and the intergenerational effect of maternal obesity, suggesting that both conditions are part of the same spectrum of endocrine perturbations.

### Limitations of the study

Given the differences between the analysis of 4 and 16-week offspring data, particularly in gene expression, including intermediate time points and additional data on protein-level changes could have provided deeper insights into the underlying phenomena and the dynamics of the transition. Furthermore, while an increasing body of literature points to an impact of paternal obesity on the metabolic trajectory of the offspring,^115^^,^^116^^,^^117^^,^^118^ this aspect was not explored in our study. Although other metabolic tissues, such as adipose tissue and muscle, are significant in metabolic physiology, our study focused solely on rhythmic liver physiology and microbiota. While 16S rRNA gene amplicon sequencing is a common method for characterizing microbiome composition, the resolution is typically at the genus level, and thereby precludes a precise assessment of the functional attributes of the communities, hence the value and utility of the metagenomic data generation and analyses included here.

## STAR★Methods

### Key resources table

**Table:** 

REAGENT or RESOURCE	SOURCE	IDENTIFIER
**Critical commercial assays**		

E.Z.N.A.® Total RNA Kit II	Omega Bio-Tek	R6934
Qiagen Gel Extraction Kit	Qiagen	28706
Illumina DNA Prep Kit	Illumina	20060059
RNA Nano 6000 Assay Kit	Agilent	5067–1511
NEBNext® UltraTM RNA Library Prep Kit	Illumina	E7770
Triglycerides content kit	Michybio	M1009A
Total cholesterol content kit	Michybio	M1010A

**Deposited data**		

Raw data of 16S rRNA sequencing and metagenomics	This paper	SRA: PRJNA881293
Raw and processed data of RNA-seq	This paper	GEO: GSE240147
GH induced genes in male GF mice	Weger et al.^66^	GEO: GSE77221
Liver gene expression in female offspring from obese mother	Savva et al.^67^	SRA: PRJNA723771
Liver gene expression in female mice treated with dihydrotestosterone, a PCOS model	Roy et al.^74^	GEO: GSE197765
Transcription factors ChIP-Atlas data	Oki et al.^119^	https://chip-atlas.org/
ChIP-seq IRF9 in male mouse macrophages	Platanitis et al.^120^	SRA: SRX4178758
ChIP-seq STAT2 in male mouse macrophages	Platanitis et al.^120^	SRA: SRX4178757
ChIP-seq SUZ12 in male mouse ES cells	Hu et al.^121^	SRA: SRX111886

**Experimental models: Organisms/strains**		

Mouse: C57BL/6J	Huafukang Biological Technology	SCXK-2020-0026

**Oligonucleotides**		

Forward_16S_rRNA: 5′-CCTAYGGGRBGCASCAG-3′	Xing et al.^122^	N/A
Reverse_16S_rRNA: 5′-GGACTACNNGGGTATCTAAT-3′	Xing et al.^122^	N/A

**Software and algorithms**		

R	R Foundation^123^	https://www.r-project.org
DESeq2	Love et al.^124^	https://doi.org/10.18129/B9.bioc.DESeq2
Enrichr	Kuleshov et al.^125^	http://amp.pharm.mssm.edu/Enrichr/
*dryR*	Weger et al.^38^	https://github.com/naef-lab/dryR
QIIME 2	Bolyen et al.^126^	https://qiime2.org/
Silva	Quast et al.^127^	http://www.arb-silva.de/
Microeco	Liu et al.^128^	https://github.com/ChiLiubio/microeco
Readfq	Chen et al.^129^	https://github.com/cjfields/readfq
MetaGeneMark	Zhu et al.^130^	http://topaz.gatech.edu/GeneMark/
MEGAHIT	Karlsson et al.^131^	N/A
CD-HIT	Li et al.^132^	http://www.bioinformatics.org/cd-hit/
Bowtie2	Langmead et al.^133^	https://sourceforge.net/projects/bowtie-bio/files/bowtie2/2.4.4/
DIAMOND	Buchfink et al.^134^	https://github.com/bbuchfink/diamond/
Hisat2	Kim et al.^135^	https://daehwankimlab.github.io/hisat2/

### Resource availability

#### Lead contact

Further information and requests for resources and reagents should be directed to and will be fulfilled by the lead contact, Frédéric Gachon (f.gachon@uq.edu.au).

#### Materials availability

This study did not generate new unique reagents.

#### Data and code availability

The RNA-Seq data have been stored in the NCBI Gene Expression Omnibus^136^ and are publicly accessible through GEO Series accession number GSE240147 (https://www.ncbi.nlm.nih.gov/geo/query/acc.cgi?acc=GSE240147). The raw data for the 16S rRNA and metagenomics data have been deposited in the NCBI Sequence Read Archive^137^ and are publicly accessible under the accession number PRJNA881293 (https://www.ncbi.nlm.nih.gov/bioproject/PRJNA881293).

This paper does not report any original code.

Any additional information required to reanalyse the data reported in this paper is available from the lead contacts upon request.

### Experimental model and study participant details

#### Mice

All animal studies were approved by the Animal Care and Ethics Committee at Peking Union Medical College Hospital (Beijing, China, XHDW-2020-041) and compliant with the National Institutes of Health guide for the care and use of laboratory animals.

As previously described,^29^^,^^138^ 4-week-old female C57BL/6 mice were purchased from Huafukang Biological Technology Co. Ltd (Beijing, China, SCXK-2020-0026). Mice were housed in a specific pathogen-free (SPF) environment. The temperature was maintained at 22 ± 2°C, and the lighting followed a 12:12 light/dark cycle (lights on at 06:00 h = zeitgeber time (ZT) 0). After a 7-day acclimation period, 5-week-old mice were randomized according to body weight into two groups: the control group (Ctr, *n* = 40) fed a standard chow diet (AIN-93G, Research Diets, 15.8 kcal% fat), and the HFD group (HFD, *n* = 40) fed a fat-rich diet (D12492, Research Diets, 60 kcal% fat). After 5 weeks on these diets, female mice were bred with 9-week-old C57BL/6 male mice, fed a control diet, in trio configuration (one male and two females) for 5 days, maintaining the allocated dietary regimen [i.e., HFD or Ctr diet]. Post-delivery, both dams and offspring were transitioned to the same control chow diet. Body weights were recorded weekly.

To investigate the effects of maternal HFD consumption before and during pregnancy on the diurnal physiology of juvenile and adult offspring, male offspring aged 4 and 16 weeks were sacrificed every 4 h (*n* = 4 per time point) over a 24-h period at six time points: 07:00 (ZT1), 11:00 (ZT5), 15:00 (ZT9), 19:00 (ZT13), 23:00 (ZT17), and 03:00 (ZT21). To minimize the influence of litter on experimental outcomes, we randomized across the six time points. Mice were sacrificed, and livers, blood serum, and cecal content were collected and snap-frozen in liquid nitrogen and stored at −80°C until further processing.

### Method details

#### Indirect calorimetry and behavior

Male offspring at 4 weeks old and 16 weeks old were randomly picked from each group. Indirect calorimetry, feeding rhythm, and locomotor activity were measured using Promethion 8-channel respirometry cages (Sable Systems International). After acclimation for 24 h to the metabolic cage, they were monitored for 2 consecutive days (24 h per day). Measurements were taken every 5 min at a 12:12 light/dark cycle. Data were averaged and graphed at 1-h intervals. Indirect calorimetry and feeding rhythm data were corrected for body weight.

#### Glucose and insulin tolerance tests

For glucose tolerance test, mice were fasted for 6 h and injected intraperitoneally with a glucose load (2 g/kg of body weight) at ZT8. For insulin tolerance test, mice were fasted for 4 h and injected intraperitoneally with insulin (Humulin R; 1.0 U/kg of body weight) at ZT6. Blood glucose levels were measured from tail bleeding before intervention and 15, 30, 60, 90, and 120 min after intervention using a glucometer (Bayer).

#### Serum biochemical parameters measurement

Blood samples were collected from the intraorbital retrobulbar plexus of mice. All blood samples were separated by centrifugation at 3,000g for 10 min at 4°C, and the serum was stored in aliquots at −80°C. Serum total cholesterol (TC), total triglyceride (TG), low-density lipoprotein cholesterol (LDL-C), high-density lipoprotein cholesterol (HDL-C), free fatty acids (FFAs), alanine transaminase (ALT), and aspartate aminotransferase (AST) were measured using chemistry analyzer (Beckman Coulter, AU5800) as previously described.^33^

#### Liver total cholesterol and triglyceride

Levels of liver TC and TG were determined using the TC (M1010A) and TG (M1009A) assay kits from Michybio. Briefly, lipids were extracted from approximately 100 mg of frozen liver tissue using 1 mL of isopropanol. Subsequently, 3–4 mm steel beads were added, and the mixture was homogenized for two rounds of 30 s each, with a 10-s pause in between, using a homogenizer (KZ-III-F, Servicebio) at 4°C. After homogenization, the mixture was centrifuged at 8,000g for 10 min at 4°C. The supernatant was then collected and used for measurements, following the manufacturer’s instructions.

#### Cecal 16S ribosomal RNA and metagenomics sequencing and analysis

16S rRNA gene amplicon sequencing and metagenomic analysis were conducted as previously described^122^ with minor modifications. Genomic DNA was extracted from the cecal contents using the cetyltrimethyl ammonium bromide (CTAB) method.^139^ The V3-V4 regions of the 16S rRNA genes were amplified using barcoded region-specific primers (341F, 5′-CCTAYGGGRBGCASCAG-3’; 806R, 5′-GGACTACNNGGGTATCTAAT-3′). The PCR products were purified using a Qiagen Gel Extraction Kit. Sequencing libraries were prepared using the TruSeq DNA PCR-Free Sample Preparation Kit (Illumina) according to the manufacturer’s instructions. Sequencing was performed on an Illumina NovaSeq platform (250 bp paired-end reads). Raw sequence reads were processed and analyzed using quantitative insights into microbial ecology (QIIME) 2 version 2022.8^126^ according to the developer’s recommendations. Sequence quality control was carried out using DADA2 algorithm and taxonomies were assigned based on the SILVA_138 library.^127^ The comparison of alpha diversity indexes between subject categories were performed using R studio with microeco package^128^ according to the developer’s operating manual, with those reads annotated as “unassigned” excluded from these specific analyses.

We utilized 1 μg of DNA as starting material from each sample to prepare the metagenomic sequencing libraries. To ensure a sufficient amount of genomic DNA for metagenomic analysis, certain cecal samples had to be reextracted. Following the manufacturer’s instructions, we employed the Next Ultra DNA Library Prep Kit for Illumina (NEB) to generate sequencing libraries. This involved sonication to fragment the DNA to approximately 350 bp, followed by end-polishing, A-tailing, and adapter ligation for Illumina sequencing. After PCR amplification, the PCR products were purified using the AMPure XP beads (Beckman-Coulter). We then assessed the size distribution of the libraries using the Agilent 2100 Bioanalyzer and quantified them via real-time PCR. Quantified libraries were equimolarly pooled and clustered on a cBot Cluster Generation System, according to the manufacturer’s protocol. Subsequent sequencing was performed on the Illumina NovaSeq platform (250 bp paired end reads).

Raw sequencing data was cleaned using Readfq (V8) with subsequent host contamination checks using Bowtie2 (version 2.2.4).^133^ Assembly of metagenome was performed using MEGAHIT (v1.0.4-beta),^131^ followed by gene prediction and abundance analysis with MetaGeneMark (V3.05)^130^ and CD-HIT (V4.5.8).^132^ Unigenes were annotated for species using DIAMOND (v0.9.9,25402007)^134^ against NCBI’s Nucleotide (non-redundant) database and for functional characteristics using Kyoto Encyclopedia of Genes and Genomes (KEGG).^140^ Differential rhythmicity analysis was performed using the *drylm* function of *dryR*.^38^ For the metagenomics data, we introduced a batch effect to account for effects caused by the re-extraction of some samples.

#### RNA extraction, sequencing, and analysis

Total RNA was extracted from liver tissues using the E.Z.N.A. Total RNA Kit II (OMEGA, R6934). Briefly, 500 μL of RNA-Solv Reagent was added to approximately 50 mg of frozen liver tissue. We then added 3–4 mm zirconia beads. This mixture was subjected to homogenization in two 30s cycles, interspersed with a 10s interval, at 4°C using a KZ-III-F homogenizer (Servicebio). Following this, 200 μL of chloroform was added, and the solution was vigorously shaken for 20s. The mixture was then allowed to settle for 3 min at room temperature before being centrifuged at 12,000g for 15 min at 4°C. The resulting RNA supernatant was collected and subsequently purified using the columns provided with the kit, in accordance with the manufacturer’s protocol. RNA integrity of all samples was assessed using the RNA Nano 6000 Assay Kit of the Bioanalyzer 2100 system (Agilent Technologies). Only RNA with a RIN above 8.0 were further processed. mRNA was purified from total RNA (3 μg of total RNA) using poly-T oligo-attached magnetic beads. Sequencing libraries were obtained using the NEBNext UltraTM RNA Library Prep Kit for Illumina following the manufacturer’s instructions.^141^ Libraries were then sequenced by the Illumina NovaSeq 6000. Raw data from published RNA-Seq studies were retrieved from NCBI GEO (accession number: GSE197765)^74^ and NCBI SRA (accession number: PRJNA723771).^67^

Reads were trimmed from adapters and mapped with Hisat2 (v2.0.5)^135^ to align the reads to the mouse reference genome (GRCm38/mm10). Uniquely mapped reads per genes were counted using FeatureCounts (v1.5.0-p3)^142^ based on the annotation from Ensembl release 94. We tested for functional enrichment using annotated gene sets from Gene Ontology (GO)^143^ using Enrichr.^125^ To statistically assess mean differences between two treatment groups we employed DESeq2,^124^ incorporating time as a covariate in the statistical model when available. We conducted differential rhythmicity analysis using *dryR*.^38^

#### Transcription factor activity analysis

Target genes of transcription factors were sourced from ChIP-Atlas^119^ for the mouse genome (mm10). Peak-calls overlapping a 5,000 bp window around a gene’s TSS were attributed to that gene. Hepatic sex-biased genes and genes that are up (GH_induced_male) or downregulated (GH_induced_male) upon injection of GH in germ-free male mice were taken from.^66^ To conduct an enrichment analysis of the genesets containing sex-specific biased genes or predicted target genes of transcription factors, we used the geneSetTest function from the limma package. This analysis was applied to gene lists that were pre-ranked defined based on results from DESeq2.^124^

### Quantification and statistical analysis

For statistical analysis, two groups with a normal distribution were assessed using the Student’s t test, while the Mann–Whitney U test was used for non-normally distributed data. When examining a 2 × 2 factorial design, we utilized a two-way ANOVA. If values matched, a repeated measures two-way ANOVA or a mixed linear model was used, complemented by the indicated post hoc test. The details of the results of the statistical analyses are available in Table S6. Differential rhythmicity analyses were performed with *dryR*^38^ using the *drylm* function for normally distributed data and the *dryseq* function for count data. Only features (e.g., gene expression profiles, ASV abundance) with a signal in ≥50% of samples per condition were included in the analysis.

The correlation matrix between serum metabolic profile measures and ASV abundance was determined using the corrplot R package. Only correlations with a Pearson coefficient that had an associated Benjamini-Hochberg adjusted P-value of less than 0.05 (determined through Fisher’s Z transform) were deemed statistically significant. We used R v.1.4.1717 with ggplot2 v 3.4.4 and GraphPad Prism version 8.0 to perform the described statistical analysis and data visualization. If not otherwise stated, data is represented as mean and error bars indicate the standard error of the mean (S.E.M).

## Supplemental information

Document S1. Figures S1–S6

Table S1. Differential rhythmicity analysis results of ASVs, related to Figure 3

Table S2. Differential rhythmicity analysis results for KEGG modules and pathways, related to Figure 4

Table S3. Differential rhythmicity analysis results for liver gene expression, related to Figure 5

Table S4. GO term enrichment analysis of differential rhythmic liver genes between maternal HFD and Ctr diet, related to Figures 5 and 6

Table S5. Transcriptional factor analysis for liver genes related to the comparison of maternal HFD and Ctr diet, related to Figure 6

Table S6. Results of the statistical analyses, related to Figures 1, 2, 3, and 6, and Supplementary Figures 1, 2,3, and 6
