# Supplementary material for: Maternal high fat diet induces circadian clock-independent endocrine alterations impacting the metabolism of the offspring
Source: iScience. 2024 Jun 21;27(7):110343. doi: 10.1016/j.isci.2024.110343 (PMC11263959; doi:10.1016/j.isci.2024.110343)
Supplement: Document S1. Figures S1–S6 [file mmc1.pdf]

iScience, Volume *27*

Supplemental information

Maternal high fat diet induces circadian clock-independent endocrine alterations impacting the metabolism of the offspring

Lu Ding, Benjamin D. Weger, Jieying Liu, Liyuan Zhou, Yenkai Lim, Dongmei Wang, Ziyan Xie, Jing Liu, Jing Ren, Jia Zheng, Qian Zhang, Miao Yu, Meltem Weger, Mark Morrison, Xinhua Xiao, and Frédéric Gachon

Supplementary Figures and Legends

Figure S1. Maternal HFD induces glucose and cholesterol metabolisms alterations in offspring, related to Figure 1

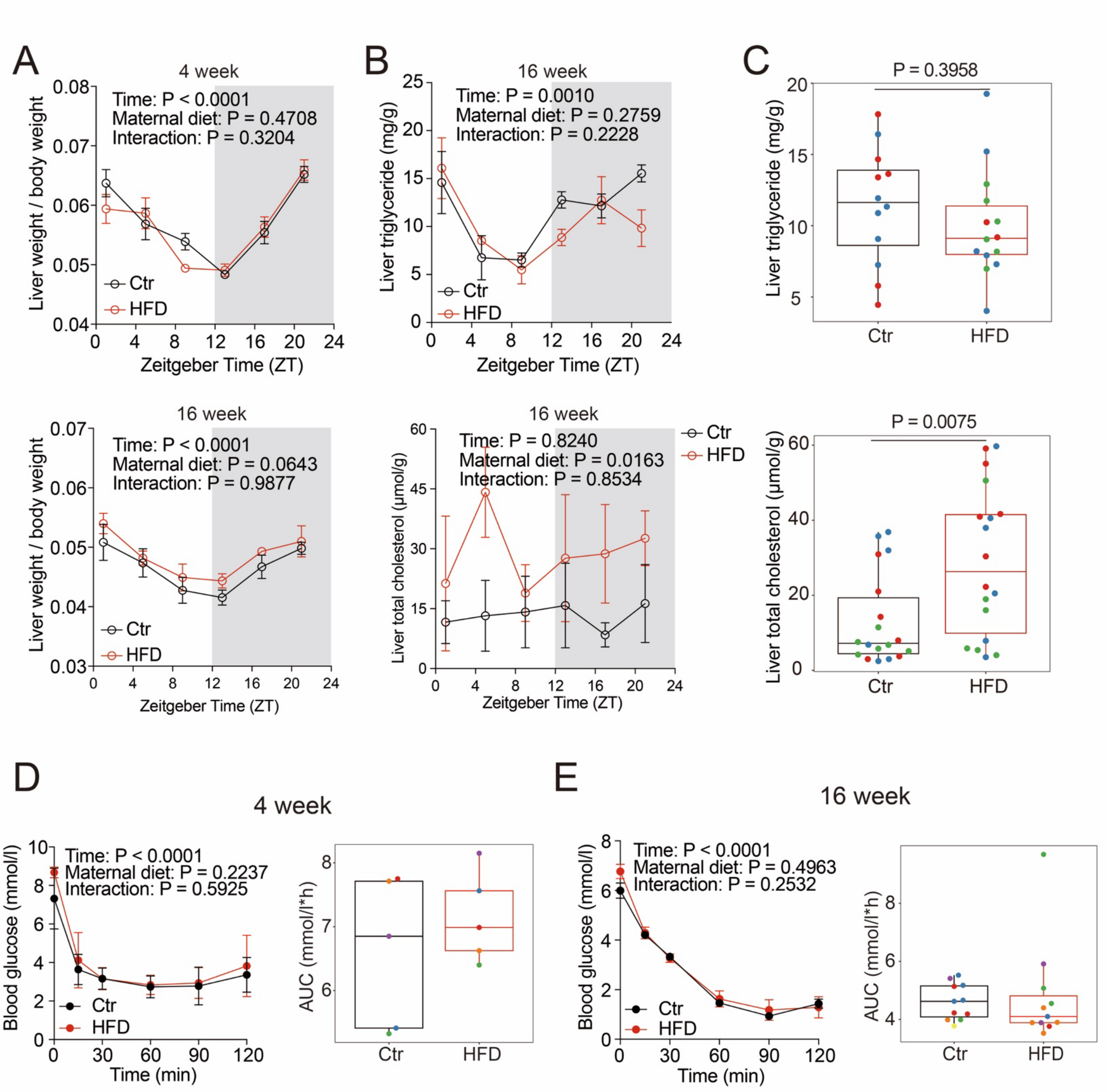

(A) Liver weight oscillation in 4-week-old offspring (top) and 16-week-old offspring (bottom). (B and C) Diurnal rhythm (B) and overall level (C) of liver triglyceride (Top) and liver total cholesterol (Bottom) in 16-week-old offspring. (D and E) Insulin tolerance test (left) and the corresponding area under the curve (right, each color represents the same litter) in 4-week-old offspring (D) and 16-week-old offspring (E). N = 2-5 mice (from different litters) per group. All boxplots are Tukey boxplots and data is assessed with a Student's t-test; line chart data are

presented as mean ± S.E.M. and are analyzed via two-way ANOVA followed by Šídák post hoc tests. The details of the statistical analysis results are available in Supplementary Table S6. Ctr: maternal control diet (black); HFD: maternal high-fat diet (red).

Figure S2. Maternal HFD does not affect the serum metabolic profiles in offspring, related to Figure 1

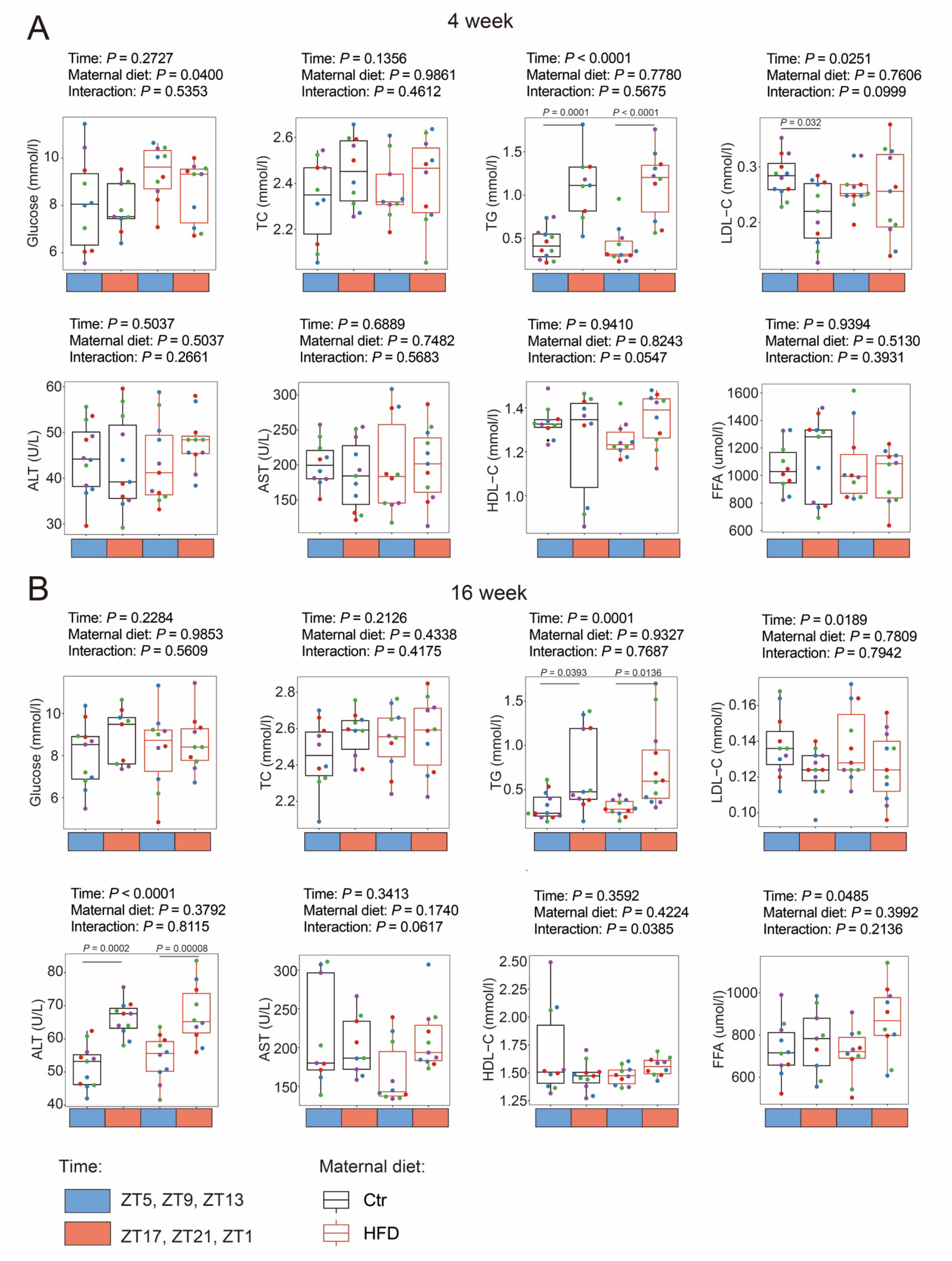

(A and B) Daily alterations of serum glucose, total cholesterol, triglyceride, LDL-C, ALT, AST, HDL-C and FFAs in 4-week-old offspring (A) and 16-week-old offspring (B). Relative levels during relative fasting (ZT5, ZT9, and ZT13) and when food has reached the cecum from feeding (ZT17, ZT21, and ZT1). Each color represents a different litter. N = 4 mice (from different litters) per group. All boxplots are Tukey boxplots and data is assessed with a two-way ANOVA followed by Šídák post hoc tests. The details of the statistical analysis results are available in Supplementary Table S6. LDL-C, low-density lipoprotein cholesterol; HDL-C, high-density lipoprotein cholesterol; FFAs, free fatty acids; ALT, alanine transaminase; AST, aspartate aminotransferase. Ctr: maternal control diet (black); HFD: maternal high-fat diet (red).

Figure S3. Effects of maternal HFD on the richness and diversity of the gut microbiota, related to Figure 3

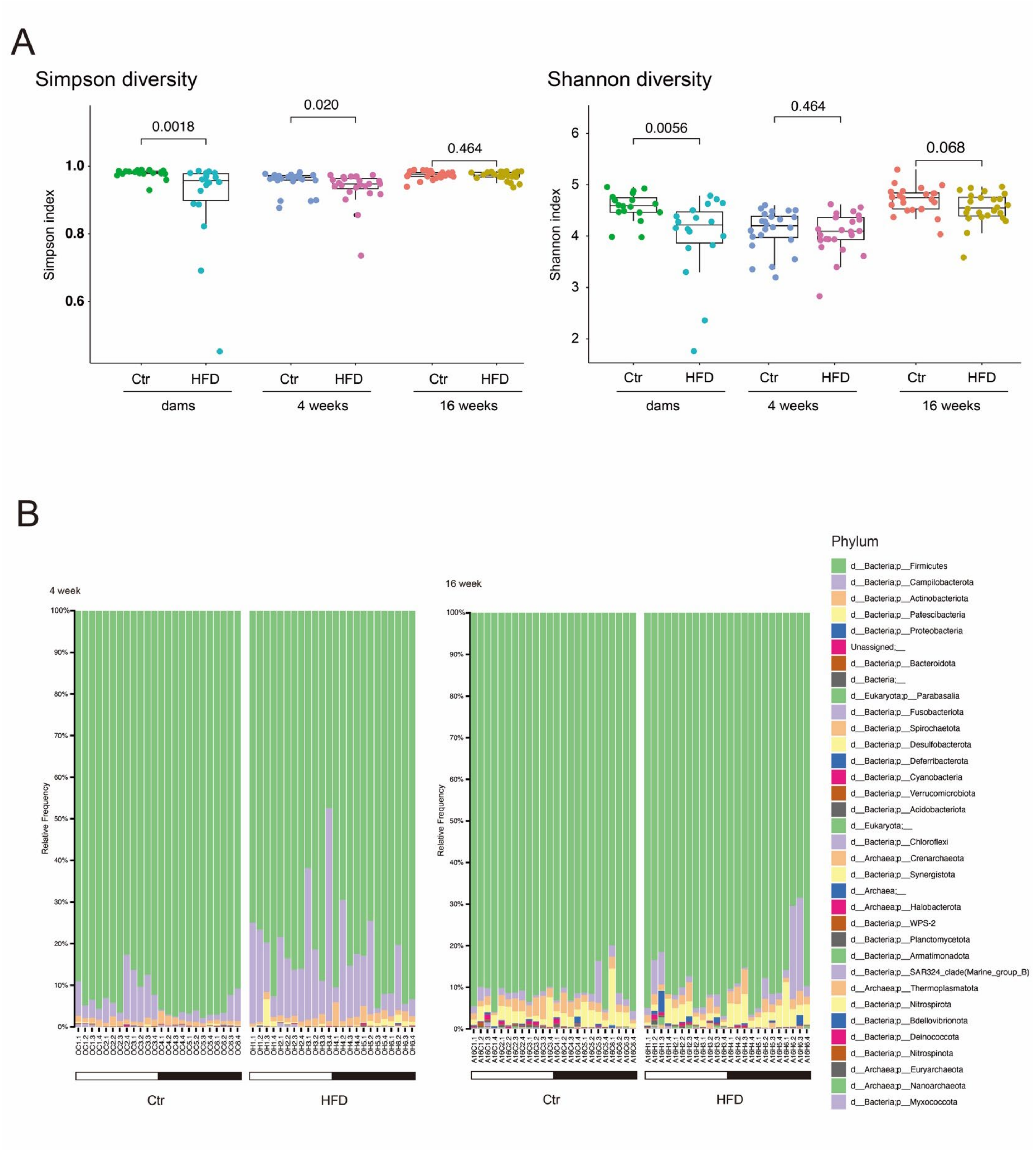

(A) Simpson (left) and Shannon (right) diversity indexes in dams, 4-week-old and 16-week-old offspring. (B) Alluvial plots of the more abundant bacteria in the phylum level in 4-week-old (left) and in 16-week-old (right)

offspring. The white and black bars denote light conditions. Boxplots are Tukey boxplots and data is assessed with a Mann-Whitney U test. The details of the statistical analysis results are available in Supplementary Table S6.

Figure S4. Effects of maternal HFD on predicted functional activity of the gut microbiota, related to Figure 4

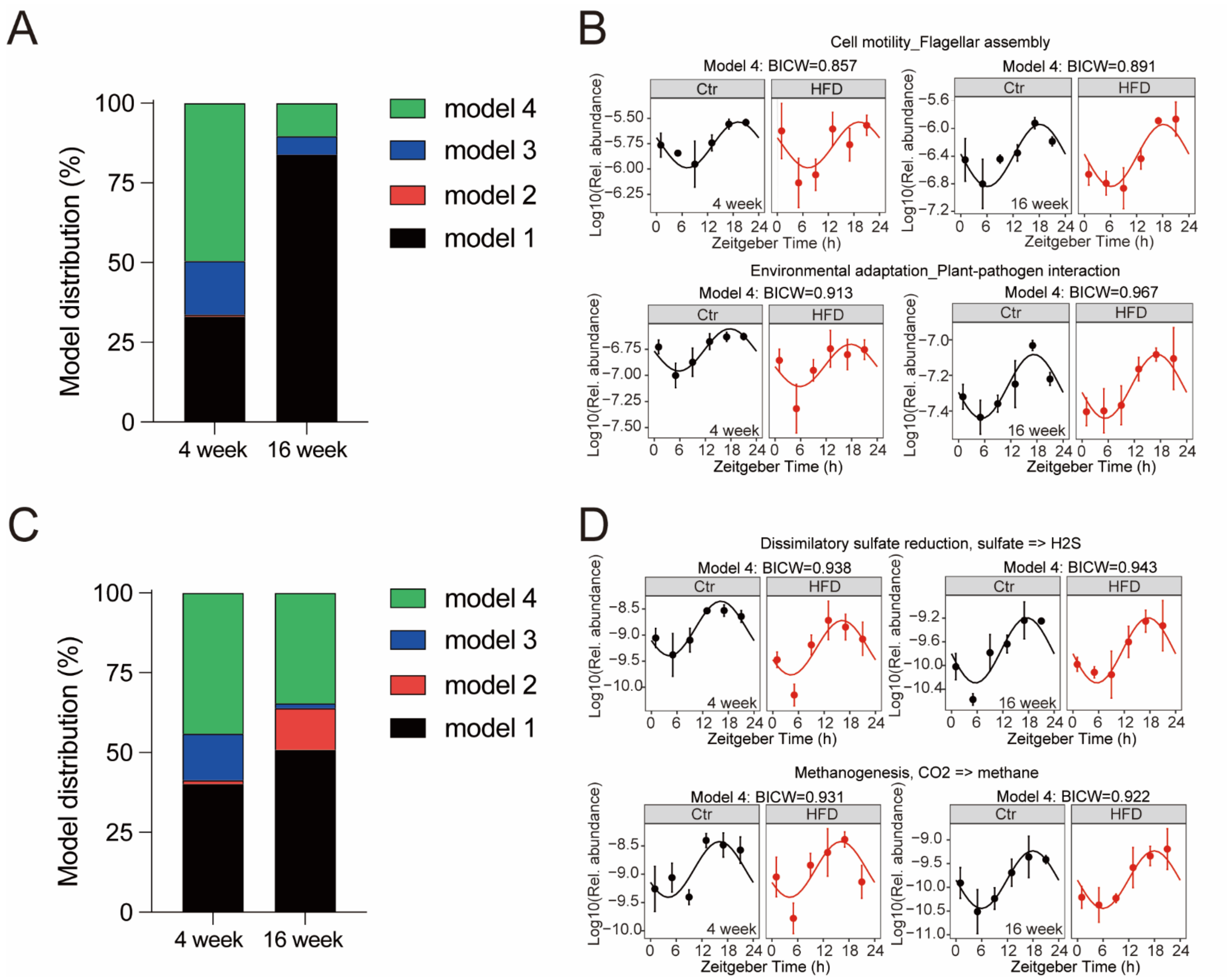

(A and C) Model distribution percentage of KEGG pathway (A) and KEGG module (C) in model 1-4. (B) Flagellar assembly pathway (top) and plant-pathogen interaction (bottom) in 4-week-old offspring (left) and 16-week-old offspring (right). (D) Dissimilatory sulfate reduction module (top) and methanogenesis module (bottom) in 4-week-old offspring (left) and 16-week-old offspring (right). The dots mark values of inferred functional activity for each Zeitgeber time (ZT) with the line illustrating the cosinor regression fit. The ZT defines the timing of entrainment by light (ZT0: lights on; ZT12: lights off). N = 3 mice (from different litters) per group. Ctr: maternal control diet (black); HFD: maternal high-fat diet (red).

Figure S5. Rhythmic alterations in liver genes of offspring subjected to maternal HFD, related to Figure 5

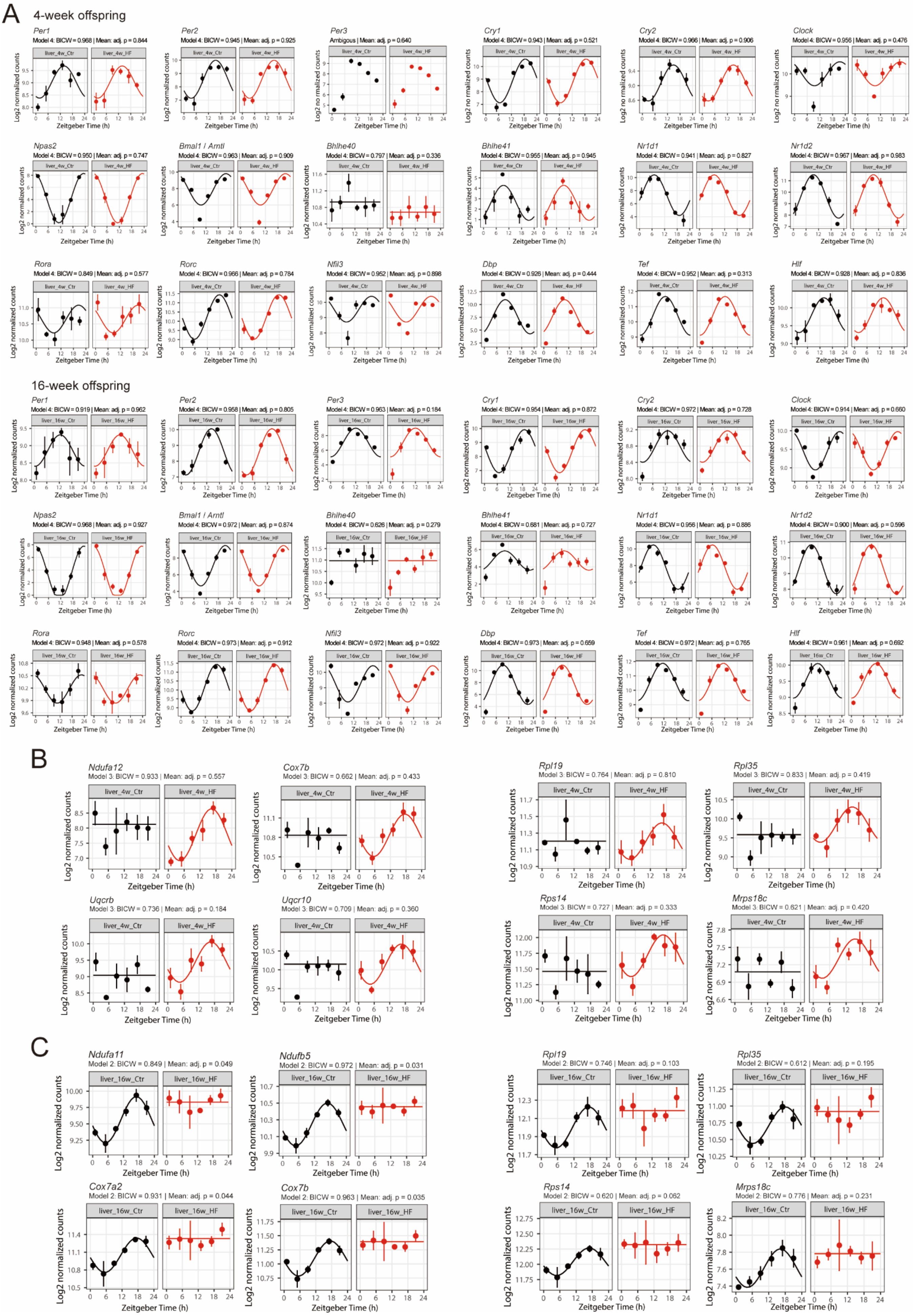

(A) Diurnal rhythm of circadian clock genes in livers of 4-week-old (top) and 16-week-old offspring (bottom). (B and C) Genes of the oxidative phosphorylation pathway (left) and ribosome pathway (right) in 4-week-old (B) and 16-week-old offspring (C) exposed to maternal HFD show rhythmic alteration. The dots mark values of log transformed relative counts of genes for each Zeitgeber time (ZT) with the line illustrating the cosinor regression fit. The ZT defines the timing of entrainment by light (ZT0: lights on; ZT12: lights off). N = 2-3 mice (from different litters) per group. Ctr: maternal control diet (black); HFD: maternal high-fat diet (red).

Figure S6. Maternal HFD induced masculinization and related interferon and mitochondrial pathway, related to Figure 6

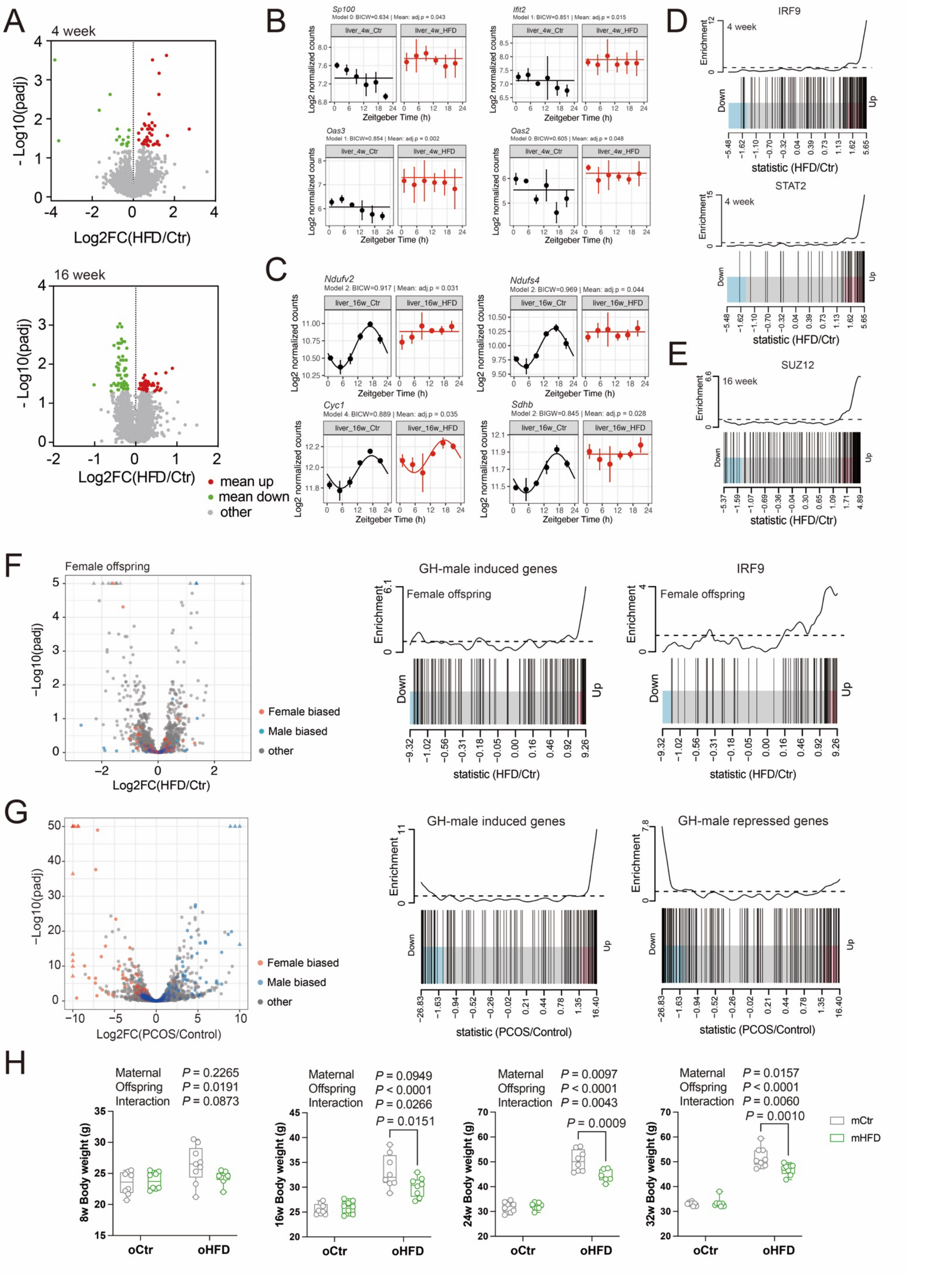

(A) Volcano plots for differentially expressed mean of genes in 4-week-old offspring (top) and 16-week-old offspring (bottom). (B) Mean up genes enriched in interferon pathway in 4-week-old offspring. (C) Mean up genes enriched in mitochondrial related pathway in 16-week-old offspring. The dots mark values of log transformed relative counts of genes for each Zeitgeber time (ZT) with the line illustrating the cosinor regression fit. Ctr: maternal control diet (black); HFD: maternal high-fat diet (red). The ZT defines the timing of entrainment by light (ZT0: lights on; ZT12: lights off). (D) Barcode plots for IRF9 (top) and STAT2 (bottom) in the liver of 4-week-old offspring with genes ordered from most up to most downregulated. (E) Barcode plots for SUZ12 in the liver of 16-week-old offspring with genes ordered from most up to most downregulated. (F) Volcano plots illustrating differentially expressed genes in the female offspring upon maternal HFD (left). Barcode plots for GH-male induced genes (middle) and IRF9 (right) in the liver of female offspring upon maternal HFD, ordering from most up to most downregulated [1]. (G) Volcano plots illustrating differentially expressed genes in the liver of PCOS female mice (left). Barcode plots for GH-male induced genes (middle) and GH-male repressed genes (right) in the liver of PCOS female mice, ordering from most up to most downregulated. Genes are ordered by t-statistics from most up to most downregulated genes. [2] (H) Offspring body weight at different weeks of age. The experiment design is shown in Figure 6E [3]. N = 2-3 mice per group. All boxplots are Tukey boxplots and data is assessed with a two-way ANOVA followed by Šídák post hoc tests. The details of the statistical analysis results are available in Supplementary Table S6.

References

1. Savva, C., Helguero, L.A., González-Granillo, M., Melo, T., Couto, D., Angelin, B., Domingues, M.R., Li, X., Kutter, C., and Korach-André, M. (2022). Molecular programming modulates hepatic lipid metabolism and adult metabolic risk in the offspring of obese mothers in a sex-specific manner. Commun Biol *5*, 1057. 10.1038/s42003-022-04022-3.
2. Roy, S., Abudu, A., Salinas, I., Sinha, N., Cline-Fedewa, H., Yaw, A.M., Qi, W., Lydic, T.A., Takahashi, D.L., Hennebold, J.D., et al. (2022). Androgen-mediated Perturbation of the Hepatic Circadian System Through Epigenetic Modulation Promotes NAFLD in PCOS Mice. Endocrinology *163*, bqac127. 10.1210/endocr/bqac127.
3. Zheng, J., Xiao, X., Zhang, Q., Yu, M., Xu, J., Qi, C., and Wang, T. (2016). The effects of maternal and post-weaning diet interaction on glucose metabolism and gut microbiota in male mice offspring. Biosci Rep *36*, e00341. 10.1042/BSR20160103.
